# *Propionibacterium freudenreichii* CIRM-BIA 129 Osmoadaptation Coupled to Acid-Adaptation Increases Its Viability During Freeze-Drying

**DOI:** 10.3389/fmicb.2019.02324

**Published:** 2019-10-09

**Authors:** Floriane Gaucher, Koffigan Kponouglo, Houem Rabah, Sylvie Bonnassie, Jordane Ossemond, Sandrine Pottier, Julien Jardin, Valérie Briard-Bion, Pierre Marchand, Philippe Blanc, Romain Jeantet, Gwénaël Jan

**Affiliations:** ^1^UMR STLO, Agrocampus Ouest, INRA, Rennes, France; ^2^Bioprox, Levallois-Perret, France; ^3^Bba, Pôle Agronomique Ouest, Régions Bretagne et Pays de la Loire, Rennes, France; ^4^Université de Rennes I, Rennes, France; ^5^CNRS, ISCR – UMR 6226, PRISM, BIOSIT – UMS 3480 Université de Rennes I, Rennes, France

**Keywords:** bacteria adaptation, freeze-drying, probiotic, starters, osmoadaptation, stress

## Abstract

*Propionibacterium freudenreichii* is a beneficial bacterium with documented effects on the gut microbiota and on inflammation. Its presence within the animal and human intestinal microbiota was correlated with immunomodulatory effects, mediated by both propionibacterial surface components and by secreted metabolites. It is widely implemented, both in the manufacture of fermented dairy products such as Swiss-type cheeses, and in the production of probiotic food complements, under the form of freeze-dried powders. The bottleneck of this drying process consists in the limited survival of bacteria during drying and storage. Protective pre-treatments have been applied to other bacteria and may, in a strain-dependent manner, confer enhanced resistance. However, very little information was yet published on *P. freudenreichii* adaptation to freeze-drying. In this report, an immunomodulatory strain of this probiotic bacterium was cultured under hyperosmotic constraint in order to trigger osmoadaptation. This adaptation was then combined with acid or thermal pre-treatment. Such combination led to accumulation of key stress proteins, of intracellular compatible solute glycine betaine, to modulation of the propionibacterial membrane composition, and to enhanced survival upon freeze-drying. This work opens new perspectives for efficient production of live and active probiotic propionibacteria.

## Introduction

Incidence of inflammatory bowel disease (IBD), of irritable bowel syndrome (IBS) and of antibiotic-associated diarrhea (ADD) is increasing in developed countries in relation with modifications of the life style. Indeed, the Western diet contains high amount of fat and proteins. The diet has an important impact on the modulation of the microbiota. A dysbiosis of the microbiota is involved in many disorders like inflammation, allergy and atopy (Moco et al., 2014; Skonieczna-Żydecka et al., 2018; Soularue et al., 2018). Ingestion of a mixture of probiotic bacteria induced or prolonged a remission in patients suffering from ulcerative colitis (Ganji-Arjenaki and Rafieian-Kopaei, 2018). In addition, the strain *Lactobacillus plantarum* 299V and *Bifidobacterium infantis* 35624 exerted healing effect on IBS (Ducrotté, 2012; Yuan et al., 2017; Sniffen et al., 2018). Probiotic are defined as “live microorganisms that, when administered in adequate amounts, confer a health benefit on the host” (FAO/WHO, 2001). Early exposition to key immunomodulatory symbiotic bacteria and or/probiotic prevent allergy and atopy (Fujimura et al., 2016; Butel et al., 2018). Bifidobacteria and propionibacteria constitute protective and early colonizing probiotic symbionts (Chang et al., 2017; Colliou et al., 2017). Furthermore, allergic diseases in Caesarean-delivered children, at 13-year follow-up, was prevented by an early exposition to a mixture of lactic acid bacteria an propionibacteria (Kallio et al., 2018).

*Propionibacterium freudenreichii* possesses the QPS (qualified Presumption of Safety EFS, European Union) and the GRAS (Generally Recognized As Safe, United States, FDA) status (Cousin et al., 2010; Rabah et al., 2018). It is consumed in high amounts due to its presence in Swiss-type cheeses, and also in probiotic food supplements. During the manufacturing, *P. freudenreichii* is responsible of the production of the beneficial short chain fatty acids acetate and propionate, the B9 (folate) and B12 (cobalamin) vitamins, and the bifidogenic compounds DHNA (1,4-dihydroxy-2-naphtoic acid) and ACNQ (2-amino-3-carboxy-1,4-naphthoquinone) (Rabah et al., 2017). Select strains of *P. freudenreichii* can modulate the gut microbiota in the colitis context and in healthy conditions (Bouglé et al., 1999; Hojo et al., 2002; Seki et al., 2004; Mitsuyama et al., 2007). This modulation depends of the production of the DHNA and ACNQ molecules which favor bifidobacteria at the expense of pathobiont Bacteroides (Isawa et al., 2002). Consumption of *P. freudenreichii* protected animals from induced intestinal disease (Foligne et al., 2010; Plé et al., 2015, 2016) and revealed healing effect on ulcerative colitis in humans (Okada, 2006; Mitsuyama et al., 2007). To optimize its effects mediated by beneficial metabolites, *P. freudenreichii* should be consumed alive. It can be consumed within Swiss-type cheese or under a powder form in tablets or capsules. The International Dairy Federation (IDF) recommends a minimum of 10^7^ live probiotic bacterial cells per gram or milliliter of production at the time of consumption (Corona-Hernandez et al., 2013).

Most of the time, probiotic bacteria are stored, transported and consumed under a powder form. Probiotics microorganisms like bacteria and yeasts are dried to produce easy-to-use preparations, which can be implemented in food, feed or pharmaceutical industry (Huang et al., 2017). Two drying processes can be used to dry bacteria: freeze-drying and spray drying. Freeze-drying is the most used process, keeps alive the largest percentage of bacteria, but it has the disadvantage of being an expensive discontinuous process (Santivarangkna et al., 2007). However, freeze-drying imposes cold and osmotic stresses (Gaucher et al., 2019b), and causes the appearance of holes in the membrane, which can lead to cell death (Carvalho et al., 2004; Giulio et al., 2005). After drying, bacteria used as probiotic will further be exposed to harsh conditions during the digestion process. Some of the probiotic actions, however, need live bacteria in the colon. Tolerance toward stomach acidity and intestinal bile salts can constitute a limit of the probiotic potential.

Bacteria adaptation increase bacteria resistance to different stresses like acid stress (Jan et al., 2002; Broadbent et al., 2010), bile salt stress (Leverrier et al., 2003, 2004), and drying (Desmond et al., 2001; Carvalho et al., 2004; Silva et al., 2005; Li et al., 2009; Paéz et al., 2012). Cross-protections provided by adaptation are generally strain-dependent (Gaucher et al., 2019b). In a previous study, osmoadaptation exerted a positive impact on *P. freudenreichii* CIRM-BIA 129 viability during freeze-drying, but not during different challenges such as heat, acid or bile salts stresses (Gaucher et al., 2019a). Concerning *P. freudenreichii* freeze-drying, almost nothing has been published. Acid adaptation and/or heat adaptation have, however, been reported to increase *Lactobacillus casei* (Paéz et al., 2012), *L. plantarum* (Paéz et al., 2012), *Lactobacillus bulgaricus* (Li et al., 2009), and *Lactobacillus reuteri* (Palmfeldt and Hahn-Hägerdal, 2000; Koch et al., 2008) viability during freeze-drying as well as *Lactobacillus paracasei* (Desmond et al., 2001), and *Lactobacillus delbrueckii* (Silva et al., 2005) viability during spray drying. During these adaptations, the membrane fluidity of the bacteria will be adjusted by the modulation of the composition of fatty acids, in particularly the amount of saturated (SFA) and unsaturated (UFA) fatty acids (Lanciotti et al., 2001; Machado et al., 2004; Streit et al., 2008; Broadbent et al., 2010). General stress protein like Clps, DnaK, GroES or GroEL are overproduced during these three adaptations (Jan et al., 2001; Leverrier et al., 2004; Savijoki et al., 2005; Dalmasso et al., 2012; Huang et al., 2016a). During osmoadaptation, *P. freudenreichii* is able to accumulate glutamate, trehalose and glycine betaine (Gaucher et al., 2019a). In hyperosmotic media, bacteria need to accumulate compatible solutes in order to preserve their turgescent pressure and to enable cell growth and division. Compatible solutes can be imported into the cell from the growth medium, or synthesized *de novo* (Csonka and Hanson, 1991). Glycine betaine is a compatible solute accumulated by different bacteria, including *Staphylococcus aureus*, *Escherichia coli*, *Enterococcus faecalis*, and *P. freudenreichii*, during osmoadaptation (Kunin and Rudy, 1991; Graham and Wilkinson, 1992; Gaucher et al., 2019a). Glutamate has already been reported as a compatible solutes accumulated during osmoadaptation (Empadinhas and Viete-Vallejo, 2008). Trehalose is a compatible solutes accumulated during acid adaptation and osmoadaptation by *P. freudenreichii* (Cardoso et al., 2007; Huang et al., 2016a; Gaucher et al., 2019a). In the present study, we combined osmoadaptation with acid or heat adaptation, in order to improve *P. freudenreichii* CIRM-BIA 129 resistance. We investigate the impact of the modulation of the fatty acid composition, compatible solutes accumulation and expression of general stress proteins, on *P. freudenreichii* tolerance toward different stresses and during freeze-drying. Combining two protective pretreatments led to enhance survival of *P. freudenreichii* upon freeze-drying.

## Materials and Methods

### Strains and Pre-culture

*Propionibacterium freudenreichii* CIRM-BIA 129 (equivalent ITG P20) was provided, stored and maintained by the CIRM-BIA Biological Resource Center (Centre International de Ressources Microbiennes-Bactéries d’Intérêt Alimentaire, INRA, Rennes, France). *P. freudenreichii* CIRM-BIA 129 was routinely cultivated in yeast extract lactate (YEL) broth (Malik et al., 1968). YEL medium contained, per liter, 21.4 g DL-lactate (60%), 10 g Yeast extract, 10 g tryptone, 0.328 g K_2_HPO_4_, and 0.056 g MnSO_4_. This leads to a non-protein nitrogen content of 14.2 g. *P. freudenreichii* was grown at 30°C without agitation under microaerophilic condition.

### Bacterial Adaptation

*Propionibacterium freudenreichii* CIRM-BIA 129 was grown under different condition: in YEL medium (0.429 osmol), or in YEL medium with 0.9 M NaCl (YEL+NaCl, 1.958 osmol) to induce osmoadaptation. Heat or acid adaptation where realized at the beginning of the stationary phase. Heat adaptation was performed by placing 25 mL (in a 50 mL Falcon tube) of *P. freudenreichii* cultures in a water bath at 42°C for 1 h (Anastasiou et al., 2006), and acid adaptation was performed by adjusting the culture at pH = 5 and then incubated during 1 h at 30°C (Jan et al., 2001).

### Stress Challenges

Heat, oxidative, bile salts and acid challenge were applied to cultures at the beginning of stationary-phase (when maximal OD was reached), or after adaptations. A strong heat challenge was performed by placing 2 mL (in a 15 mL Falcon tube) of *P. freudenreichii* culture in a water bath at 60°C for 10 min (Leverrier et al., 2004). A lower heat stress was performed by placing 2 mL (in a 15 mL Falcon tube) of *P. freudenreichii* culture in a water bath at 55°C for 30 min (Leverrier et al., 2004). Oxidative challenge was applied by adding 1.25 mM of hydrogen peroxide (Labogros, France) to 2 mL of *P. freudenreichii* culture during 1 h at 30°C (Serata et al., 2016). Acid challenge was applied by re-suspending *P. freudenreichii* in MMO medium adjusted to pH 2.0 by using HCl at 30°C followed by a 1 h incubation (Jan et al., 2000). Bile salts challenge was performed by adding 1 g.L^–1^ of a bile salts mixture (an equimolar mixture of cholate and deoxycholate; Sigma Chemical, St. Louis, MO, United States) in the culture during 1 h at 37°C (Leverrier et al., 2003). CFU counting was performed after challenge. In order to calculated survival percentage, a CFU counting was made, with untreated culture left for the same time at 30°C as a control.

### Freeze-Drying

*Propionibacterium freudenreichii* CIRM-BIA 129 were grown in the YEL and YEL+NaCl medium and with or without acid or heat adaptation. At the beginning of the stationary phase, cultures were harvested (8000 g, 10 min, 30°C). Pellets were then homogenized in a maltodextrin solution (100 g.L^–1^) (Roquette, France). The bacterial solutions were then freeze-dried (2253-04, Serail, France).

### Fatty Acids Analysis

*Propionibacterium freudenreichii* CIRM-BIA 129 was grown in YEL and YEL+NaCl medium with or without acid or heat adaptation at the beginning of the stationary-phase. Cells were then washed with sterile distilled water. Cells were harvested by centrifugation (8000 *g*, 15 min). Saponification was performed by adding 3 mL of sodium methoxide (3.75 M in methanol) (Sigma Chemical, St. Louis, MO, United States) and shaking vigorously for 10 s and incubated during 25 min at 100°C. Samples were cooled to performed methylation, a solution of HCL and methanol were added (HCl 3.5 M and methanol 42% final concentration), samples were vortexed 10 s and incubated during 10 min at 80°C. Before the extraction of BAME (bacterial acid methyl ester) samples were cooled on ice and a solution of hexane and diethyl ether (hexane 50% and diethyl ether 50%) was added. Samples were agitated during 10 min and then decanted, aqueous phases were removed before organic phases washing with NaOH 3 M solution. Samples were then agitated and decanted. Organic phases were then collected for analysis.

The analyses were performed on an Agilent gas chromatograph (7890A) equipped with a BPX70 capillary column (120 m × 0.25 mm × 0.25 μm, SGE, Victoria, Australia) and coupled to a flame ionization detector (Agilent Technologies, Les Ulis, France). Hydrogen was used as carrier gas, and the injection volume was 0.5 μL. Injection was done by a cool on column injector. Detection temperature was 250°C. Agilent MSD ChemStation software was used for data acquisition. Components were identified from the retention time measured from BAME (Bacterial Acid Methyl Ester CP) standards (Merck, France).

Results were expressed as relative percentages of each fatty acid, which were calculated as the ratio of the surface area of the considered peak to the total area of all peaks. The ratio of unsaturated to saturated fatty acids (U/S) was determined. Analyses were made in triplicate.

### Identification and Quantification of Osmoprotectants Accumulated by *P. freudenreichii* CIRM-BIA 129

#### Extraction of Accumulated Osmoprotectants

*Propionibacterium freudenreichii* CIRM-BIA 129 was grown in YEL and YEL+NaCl medium with or without acid adaptation at the beginning of the stationary-phase. During exponential phase (OD = 0.8), cells were harvested by centrifugation (8000 *g* 10 min). Cells were washed twice in a NaCl solution with the same osmolarity than the culture medium. Cells were then re-suspended in 2 mL of distilled water, then 8 mL of absolute ethanol were added. The suspension was homogenized and centrifuged (8000 *g*, 10 min) in order to remove cell fragments. The supernatant extract was evaporated during 7 h with a rotary evaporator. Dried extracts were then solubilized in deuterium oxide (Sigma-Aldrich, United States).

### NMR (Nuclear Magnetic Resonance) Analyses

All ^1^H and ^13^C NMR spectra were recorded at 298 K on a Bruker Avance 500 spectrometer equipped with a 5 mm TCI triple-resonance cryoprobe (PRISM core facility, Rennes). ^1^H spectra were acquired with a 6 kHz spectral width, 32 K data points and a total repetition time of 6.73 s. ^13^C spectra were acquired using a proton power-gated decoupling sequence with a 30° flip angle, a 30 kHz spectral width, 64 K data points and a total repetition time of 3.08 s. The data were processed with Topspin software (Bruker Biospin). Before applying the Fourier transform, free induction decays of ^1^H spectra were treated with an exponential broadening of 0.3 Hz.

Samples were solubilized in D_2_O. 3-(Trimethylsilyl) propionic-2,2,3,3-d4 acid sodium salt (TSP-d4) (Sigma-Aldrich, United States) served as an internal reference for ^1^H and ^13^C chemical shifts. The relative concentration of trehalose, glutamate and glycine betaine in the samples was determined by the integration of the peaks’ areas of their ^1^H signals relative to the internal standard TMSP. Results are expressed as NMR relative units (RU).

### Label Free Proteomics

#### Whole-Cell Protein Extraction and Protein Tryptic Digestion

The label free proteomics has been conducted as described by Gaucher et al. (2019a). At the beginning of stationary phase or after acid adaptation, *P. freudenreichii* cells were harvested by centrifugation and washed twice with PBS buffer (NaCl 8 g.L^–1^, KCl 2 g.L^–1^ KH_2_PO_4_ 2 g.L^–1^, Na_2_HPO_4_ 12H_2_O 35,8 g.L^–1^). Briefly, cells pellets were washed, and cells were resuspended in a lysis solution. The solution was sonicated and cells were broken using zirconium beads in a homogenizer. Proteins were harvested, cleaned and quantified before digestion and protein were extracted before digestion. Tryptic digestion was performed on 100 μg of whole-cell proteins from each sample during 15 h at 37°C using Sequencing Grade Modified Trypsin (Promega, Madison, WI, United States) according to the manufacturer’s instructions and as described previously (Huang et al., 2018). Spectrophotometric-grade trifluoroacetic acid (TFA) (Sigma-Aldrich, United States) was added in order to stop the digestion.

#### Nano-LC-MS/MS

Experiments were performed as previously described (Huang et al., 2018). Briefly, experiments were performed using a nano RSLC Dionex U3000 system fitted to a Q-Exactive mass spectrometer (Thermo Fisher Scientific, San Jose, CA, United States) equipped with a nano-electrospray ion source. Peptides separation was performed on a reversed-phase column (PepMap 100 C18, 75 μm i.d., 250 mm length, 3 μm particle size, 100 Å pore size; Dionex, Amsterdam, Netherlands) and The spectra of eluted peptides were recorded in full MS mode and selected in a mass range 250–2000 m/z for MS spectra with a resolution of 70,000 at m/z 200. For each scan, the ten most intense ions were selected for fragmentation. MS/MS spectra were recorded with a resolution of 17,500 at m/z 200 and the parent ion was subsequently excluded from MS/MS fragmentation for 20 s. The instrument was externally calibrated according to the supplier’s instructions.

#### Protein Identification

Protein identification was performed as previously described (Huang et al., 2018). Peptides were identified from the MS/MS spectra using X!Tandem pipeline software (Langella et al., 2017). The search was performed against the proteome of strain *P. freudenreichii* CIRM-BIA 129 (ITG P20) (downloaded from NCBI.nlm.nih.gov on the 23rd of August 2018). The strain is registered as “*P. freudenreichii* subsp. *freudenreichii* ITG P20” (GenBank: CCBE000000000.1, BioProject PRJEB4826). Database search parameters were specified as follows: trypsin cleavage was used and the peptide mass tolerance was set to 10 ppm for MS and 0.05 Da for MS/MS. Oxidation of methionine and phosphorylation of threonine, serine and tryptophan were selected as a variable modification. For each peptide identified, a minimum score corresponding to an *e*-value below 0.05 was considered as a prerequisite for peptide validation.

#### Protein Quantification

Protein quantification was performed as previously described (Huang et al., 2018). Every peptide identified by tandem mass spectrometry was quantified using the free MassChroQ software (Valot et al., 2011) before data treatment and statistical analysis within the R software (R 3.2.2, Project for statistical computing). A specific R package called “MassChroqR” was used to automatically filter dubious peptides for which standard deviation of retention time was superior to 30 s and to regroup peptide quantification data into proteins. For peak counting analysis, variance analysis was performed on proteins with a minimum peak ratio of 1.5 between both culture conditions. Proteins with an adjusted *p*-value < 0.05 were considered significantly different.

For XIC based quantification, normalization was performed to take into account possible global quantitative variations between LC-MS runs. Peptides shared between different proteins were automatically excluded from the data set as well as peptides present in less than 85% of samples. Missing data were then imputed from a linear regression based on other peptide intensities for the same protein (Blein-Nicolas et al., 2015). Analysis of variance was used to determine proteins with significantly different abundance between our two culture conditions.

### SDS-PAGE and Western-Blotting

The extraction, the SDS-Page and the Western blot have been conducted as Rabah et al. (2018). Briefly, *P. freudenreichii* was grown in YEL or YEL+NaCl with or without acid or heat adaptation. Cells were harvested by centrifugation, washed two times with PBS. After centrifugation, supernatants were removed and lysis solution (Tris–HCl 50 mM pH 7.5, SDS 0.3%, DTT200 mM) was added to bacterial pellets and samples were frozen prior to sonication and cell lysis using Precellys^®^ Evolution homogenizer. Resulting SDS extracts were recovered by centrifugation (21,000 × *g*, 4°C, 20 min) and analyzed by one-dimensional electrophoresis SDS-PAGE. The protein extracts from triplicate experiments were pooled and the protein amount was determined using 2-D Quant Kit (GE Healthcare). Protein extracts were separated by 12% SDS–PAGE and then transferred to PVDF membranes (GE Healthcare). A solution with 3% non-fat dry milk diluted in TBS (Tris 10 mM, NaCl 0.15 M, 0.3% tween 20) was added on the PVDF membranes to perform locking. Membranes were washed and then incubated over-night at 4°C with anti-SlpB primary antibodies purified from rabbit sera (AGRO-BIO, France) at the dilution 1:10,000. After washing, membranes were incubated with secondary antibodies: anti-rabbit IgG conjugated with horseradish peroxidase (1:15,000, AGRO-BIO, France) for 2 h at room temperature. Bound antibodies were visualized with ECL Plus system (GE Healthcare, Vélizy, France) and blots were scanned using the Syngene GBox (Ozyme, Saint-Quentin-en-Yvelines, France).

### Statistical Analysis

The data were from triplicate samples. All the results are presented as mean value with standard deviation. Statistical analyses using one-way ANOVA with Tukey *post hoc* analyses for multiple comparison. Calculations were performed using GraphPad Prism Software (Prism 7 for Windows).

## Results

### *P. freudenreichii* Viability During Challenges and Freeze-Drying

**FIGURE 1:**
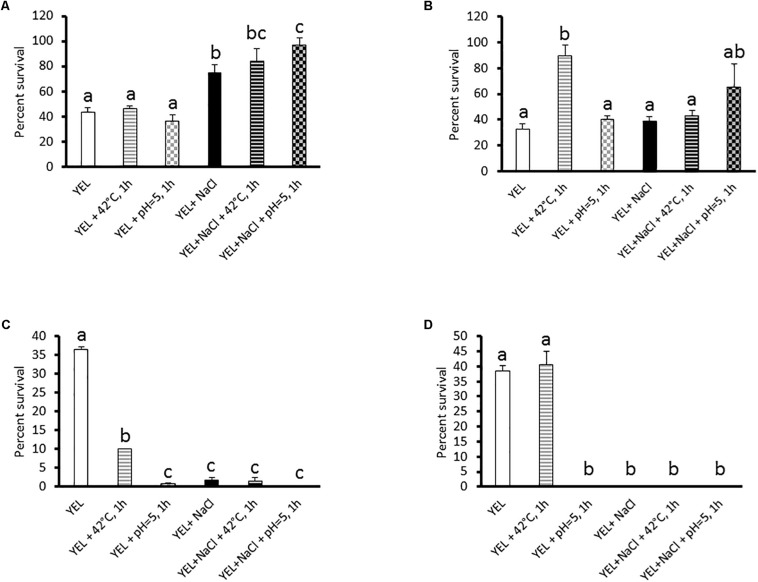
Viability of *Propionibacterium freudenreichii* CIRM-BIA 129 after freeze-drying **(A)**, heat **(B)**, acid **(C)**, and bile salts **(D)** challenges. The strain was previously grown in YEL and YEL+NaCl broths, and subjected to heat and acid adaptation. Errors bars represent the standard deviation for triplicate experiments. Significances differences are reported with different letters above columns (*p* > 0.05). YEL: Yeast Extract Lactate. 42°C, 1 h: thermo-adaptation at 42°C during 1 h. pH = 5, 1 h: acid-adaptation at pH = 5 during 1 h.

We subjected *P. freudenreichii* to freeze-drying, heat, acid, and bile salts stress challenges, in order to monitor its stress tolerance. *P. freudenreichii* CIRM-BIA 129 viability was determined by numeration (CFU counting) before and after these challenges, which were selected as being relevant to freeze-drying process, Swiss-type cheese making, and to digestive constraints. As shown in Figure 1A, addition of salt (0.9 M NaCl) to the YEL growth medium increased *P. freudenreichii* CIRM-BIA 129 viability during freeze-drying (43 to 74.4%), yet showing no effect on its viability during heat lethal challenge (55°C, 30 min) (Figure 1B). This hyperosmotic constraint even had a negative impact on *P. freudenreichii* CIRM-BIA 129 survival during acid (pH 2, 1 h, 36.4 to 1.8%) and bile salt (1 g.L^–1^, 1 h, 38.3 to 0%) challenges (Figures 1C,D). Osmoadaptation, acid adaptation, and the combination thereof led to a viability below the detection threshold during bile salts challenge. A sublethal heat pretreatment (42°C, 1 h) had *per se* a positive impact on survival during a subsequent heat lethal challenge (Figure 1B, 32.5 to 87.6%), yet a negative impact on survival during acid challenges (Figure 1C, 36.4 to 10%, respectively). Acid adaptation (pH 5, 1 h) *per se* did not enhance tolerance, neither toward these lethal challenges, nor toward freeze-drying. Heat adaptation, or acid adaptation, combined to osmoadaptation, had a negative or no impact on *P. freudenreichii* CIRM-BIA 129 tolerance toward stress challenges drying (Figures 1B–D). However, such a combination enhanced *P. freudenreichii* CIRM-BIA 129 survival during freeze-drying (Figure 1A, 43 to 90.5 and 96.7%, respectively). Best viability during freeze-drying was obtained for *P. freudenreichii* CIRM-BIA 129 grown in YEL+NaCl, with an acid-adaptation at the beginning of the stationary phase. Further analyses were then performed on *P. freudenreichii* CIRM-BIA 129, grown in YEL or in YEL+NaCl, with or without acid or heat adaptation, in order to elucidate mechanism responsible for enhanced tolerance toward freeze-drying.

### Membrane Fatty Acids Composition Is Modulated by Acid, Heat and Osmoadaptation

**FIGURE 2:**
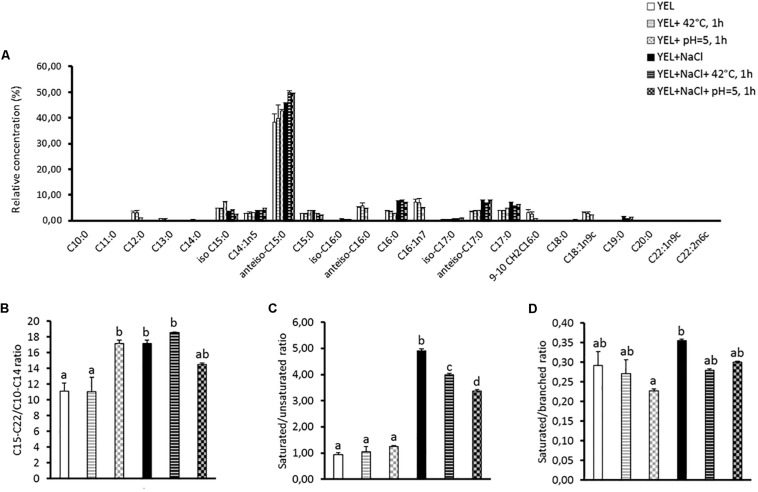
Membrane fatty acids (% of total membrane fatty acids) of *Propionibacterium freudenreichii* CIRM-BIA 129 in different conditions. The fatty acids composition **(A)**, the saturated/unsaturated fatty acids ratios (SFA/UFA) **(B)**, the saturated/branched fatty acids ratio (SFA/BFA) **(C)** and the C15-C22/C10-C14 ratio **(D)** are shown for the strain grown in YEL and YEL+NaCl broths and subjected to heat and acid adaptation. Error bars represent the standard deviation for triplicate experiments. Significant differences are reported with different letters above the columns (*p* > 0.05). YEL: Yeast Extract Lactate. 42°C, 1 h: thermo-adaptation at 42°C during 1 h. pH = 5, 1 h: acid-adaptation at pH = 5 during 1 h.

The fatty acid composition of membrane lipids was analyzed to better understand *P. freudenreichii* CIRM-BIA 129 adaptation to high osmotic pressure, to thermal stress or to acidification. As shown in Figure 2, a total of 23 fatty acids were detected in *P. freudenreichii* CIRM-BIA 129 membrane, using gas chromatography, reported in Supplementary Table S1. This includes saturated, unsaturated and branched fatty acids. *P. freudenreichii* CIRM-BIA 129 contains a high proportion of branched fatty acids (52.37–62.15%), and lower amounts of saturated (13.54–18.30%), and unsaturated ones (4.28–16.45%). The anteiso C15:0 was detected as the major fatty acid, which proportion was furthermore highly modulated during *P. freudenreichii* CIRM-BIA 129 adaptations (Figure 1A). Osmoadaptation increased the amount of long fatty acids (anteiso C17:0, C17:0, and C19:0) and decreased the amount of short fatty acid (C12:0 and C13:0). Accordingly, the C15-C22/C10-C14 ratio was enhanced as a result of osmoadaptation and of acid adaptation (Figure 2B). The amount of C16;1n7 and C18:1n9c decrease sharply for the bacteria grown in salty medium, by contrast the saturated fatty acid C16:0 and C17:0 are increasing during osmoadaptation (Supplementary Table S1). Such variations led to enhanced saturated/unsaturated ratio upon osmoadaptation (Figure 2C). Heat or acid-adaptation, applied after osmoadaptation can modulate this ratio. Saturated and branched fatty acid proportions are modulated in the different conditions, but the ratio saturated/branched did not show significant variations (Figure 2D).

### Osmoprotectants Accumulation Is Modulated by the Acid and Osmoadaptation

**FIGURE 3:**
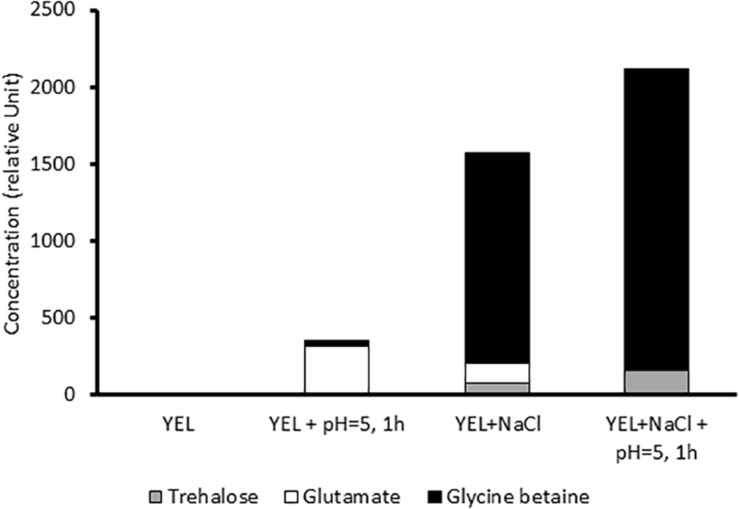
Compatible solutes accumulation (relative units) after growth in YEL and YEL+NaCl broths, with and without acid adaptation.

When cultivated in the isotonic and rich YEL growth medium in the absence of NaCl and of acid or heat stress, no trehalose, no glutamate and no glycine betaine was detected in the cytoplasm of *P. freudenreichii* CIRM-BIA 129 (Figure 3). However, acid adaptation, at the end of growth, at the beginning of stationary phase, triggered glutamate accumulation in the same medium. During growth in rich YEL medium in the presence of NaCl, *P. freudenreichii* CIRM-BIA 129 mainly accumulated glycine betaine, as well as lower amounts of glutamate and of trehalose. In these osmoadapted cultures, acid adaptation at the beginning of the stationary phase further increased accumulation of glycine and of trehalose, at the expense of glutamate.

### Modulation of Protein Expression During Osmoadaptation and Acid Adaptation

**FIGURE 4:**
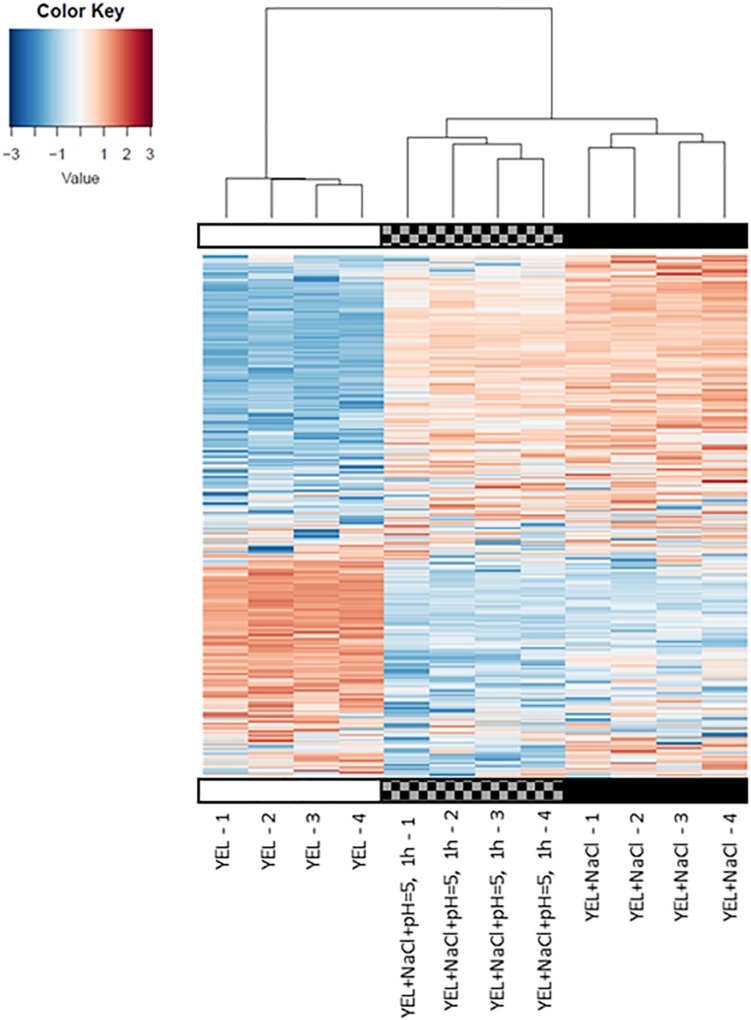
Effects of osmoadaptation and acid-adaptation on *P. freudenreichii* CIRM-BIA 129 proteome.

To understand the respective impact of *P. freudenreichii* CIRM-BIA 129 osmoadaptation and of osmoadaptation coupled to acid adaptation, we realized a proteomic differential analysis. Theoretically, 2176 proteins are encoded by *P. freudenreichii* CIRM-BIA 129 genome (GCA_000723545.1). In this study, a total of 857 proteins were detected using mass spectrometry. This represents a coverage of 39%. Proteomics data have been deposit into INRA data base (Jardin, 2019). We focused on proteins that were differentially abundant for at least one condition between YEL, YEL+NaCl, and YEL+NaCl+pH = 5, 1 h, with a minimum ratio of 1.5 or less than 0.66. A total of 216 proteins were significantly modulated, 208 between YEL and YEL+NaCl (see Supplementary Table S2) and eight proteins between YEL+NACl and YEL+NaCl+pH = 5, 1 h (Table 1). As shown in Figure 4, the heat map indicated that the two proteomes corresponding to, respectively, YEL and YEL+NaCl, were very different from the control one corresponding to YEL. By contrast, the proteome corresponding to YEL+NACl+pH = 5, 1 h is very closed to that of bacteria grown in YEL+NaCl. Salt addition strongly affected *P. freudenreichii* CIRM-BIA 129 proteome, while acid adaptation after osmoadaptation modulated the expression of few proteins. More precisely, 20 proteins modulated during osmoadaptation were involved in amino acids metabolism and transport, 17 proteins in carbohydrate transport and metabolism, five proteins in lipid transport and metabolism, six proteins in cell wall-membrane-envelope biogenesis. Another set of 17 proteins belonged to the “post-translational modification, protein turnover and chaperones” category (Table 2). All the other modulated proteins are presented in Supplementary Table S2.

**TABLE 1:** Proteins modulated during acid-adaptation after osmoadaptation. Proteins were determined by using a database composed of proteome of *P. freudenreichii* CIRM-BIA 129 (downloaded from NCBI.nlm.nih.gov 2018).

**Accession**	**Description**	**YEL+NaCl+ pH = 5/YEL+NaC ratiol**
**Energy production and conversion**		
emb|CDP49838.1|	Sulfite reductase [ferredoxin]	0.53
emb|CDP49142.1|	L-lactate permease	0.49
**Amino acid transport and metabolism**		
emb|CDP49535.1|	Cysteine synthase 2	0.49
**Replication, recombination and repair**		
emb|CDP49216.1|	Putative endonuclease III	0.61
**Inorganic ion transport and metabolism**		
emb|CDP49684.1|	ABC-type transport systems, periplasmic component	0.58
emb|CDP48302.1|	Heavy metal transport/detoxification protein	0.54
**Secondary metabolites biosynthesis, transport, and catabolism**		
emb|CDP49310.1|	Dioxygenase	0.64
**Function unknown**		
emb|CDP49347.1|	Uridine phosphorylase	0.52

*Ratio were calculated using the XIC methods and indicate induction (ratio > 1.5) or repression (ratio > 0.66) by the acid adaptation.*

**TABLE 2:** Proteins modulated during osmoadaptation belonging to the COG category “amino acid metabolism and transport,” “carbohydrate transport and metabolism,” “lipid transport and metabolism” and “cell wall-membrane-envelope biogenesis.”

**Accession**	**Description**	**YEL+NaCl/YEL ratio**
**Amino acid transport and metabolism**		
emb|CDP48583.1|	Prephenate dehydrogenase	2.90
emb|CDP47878.1|	ATP-binding protein opuCA of Glycine betaine/carnitine/choline ABC transporter	2.22
emb|CDP48936.1|	Aminopeptidase	2.02
emb|CDP48564.1|	polar amino acid ABC transporter, ATP binding component	1.86
emb|CDP47695.1|	Nucleoside-diphosphate kinase	1.82
emb|CDP48687.1|	Glycine cleavage H-protein (lipoate-binding)	1.79
emb|CDP48582.1|	Cytidylate kinase (CK) (Cytidine monophosphate kinase) (CMP kinase)	1.78
emb|CDP49472.1|	ABC-type choline/glycine betaine transport, ATP-binding protein	1.67
emb|CDP49173.1|	Dihydroxy-acid dehydratase	1.65
emb|CDP49146.1|	L-asparaginase I	1.65
emb|CDP48643.1|	Tryptophan synthase alpha chain (TrpA)	1.55
emb|CDP47760.1|	Kinase ArgK	1.52
emb|CDP49589.1|	Acetylornithine deacetylase/Succinyl-diaminopimelate desuccinylase related deacylase	1.51
emb|CDP47866.1|	Serine acetyltransferase	1.51
emb|CDP48503.1|	Amidohydrolase (Peptidase M20D) (Putative metal-dependent amidase/aminoacylase/carboxypeptidase)	0.67
emb|CDP48372.1|	Binding protein of oligopeptide ABC transporter (OPN: undef: Oligopeptides)	0.65
emb|CDP49606.1|	Shikimate 5-dehydrogenase	0.64
emb|CDP49535.1|	cysteine synthase 2	0.54
emb|CDP49386.1|	Phospho-2-dehydro-3-deoxyheptonate aldolase	0.50
emb|CDP47931.1|	Solute binding protein of the ABC transport system	0.49
**Carbohydrate transport and metabolism**		
emb|CDP49182.1|	iolB (Myo-inositol catabolism IolB protein)	2.04
emb|CDP49834.1|	Ribose-5-phosphate isomerase 3	1.90
emb|CDP48739.1|	Phosphoketolase pyrophosphate	1.87
emb|CDP48882.1|	Phosphoglycerate mutase/fructose-2,6-bisphosphatase	1.84
emb|CDP49742.1|	Endonuclease	1.68
emb|CDP48781.1|	Alpha-glucan phosphorylase	1.63
emb|CDP48893.1|	Oxidoreductase	1.62
emb|CDP47720.1|	6-phosphogluconate dehydrogenase, decarboxylating	1.56
emb|CDP49513.1|	Alpha-1,4-glucosidase	1.55
emb|CDP49568.1|	Polyphosphate glucokinase	0.66
emb|CDP48902.1|	Fructose-bisphosphate aldolase class I	0.63
emb|CDP49639.1|	Gluconate kinase (Gluconokinase)	0.59
emb|CDP47629.1|	Glycogen debranching enzyme GlgX	0.49
emb|CDP47859.1|	Binding protein of ribose ABC transporter	0.46
emb|CDP48824.1|	Dihydroxyacetone kinase	0.43
emb|CDP49430.1|	Glucose-1-phosphate adenylyltransferase (ADP-glucose synthase) (ADP-glucose pyrophosphorylase) (ADPGlc PPase)	0.39
emb|CDP49099.1|	PTS system, mannose/fructose/sorbose family, IIA component subfamily	0.26
**Lipid transport and metabolism**		
emb|CDP49767.1|	Inositol-1-phosphate synthase	1.87
emb|CDP47827.1|	Enoyl-CoA hydratase/carnithine racemase CaiD	1.84
emb|CDP49388.1|	Acyltransferase PlsC	1.52
emb|CDP48433.1|	Acyl carrier protein (ACP)	0.59
emb|CDP48431.1|	Carboxylic ester hydrolase	0.57
**Cell wall/membrane/envelope biogenesis**		
emb|CDP47662.1|	GTP-binding protein LepA	2.32
emb|CDP49368.1|	UDP-N-acetylmuramoyl-tripeptide–D-alanyl-D-alanine ligase (UDP-MurNAc-pentapeptide synthetase) (D-alanyl-D-alanine-adding enzyme)	1.98
emb|CDP49372.1|	S-adenosyl-L-methionine-dependent methyltransferase mraW	1.97
emb|CDP49370.1|	Cell division protein FtsI (penicillin-binding protein 2) (Peptidoglycan glycosyltransferase)	1.88
emb|CDP47743.1|	UDP-N-acetylmuramyl tripeptide synthase (Mur ligase)	1.76
emb|CDP49363.1|	UDP-N-acetylmuramate–L-alanine ligase (UDP-N-acetylmuramoyl-L-alanine synthetase)	1.55
**Post-translational modification, protein turnover, and chaperones**		
emb|CDP48339.1|	Heat shock protein 20 3 (20 kDa chaperone 3)	3.25
emb|CDP49617.1|	Thiol peroxidase	2.50
emb|CDP49795.1|	Thioredoxin	2.49
emb|CDP49065.1|	Surface layer protein A (S-layer protein A)	2.24
emb|CDP49048.1|	Thioredoxine	2.15
emb|CDP49400.1|	HesB protein	1.92
emb|CDP48411.1|	SmpB SsrA-binding protein	1.92
emb|CDP48424.1|	Heat shock protein 20 2 (20 kDa chaperone 2)	1.68
emb|CDP48051.1|	Protein GrpE 2 (HSP-70 cofactor 2) (Co-chaperone protein GrpE2)	1.62
emb|CDP49021.1|	Protein GrpE 1 (HSP-70 cofactor 1) (Co-chaperone protein GrpE1)	1.61
emb|CDP48340.1|	Heat shock protein 20 1 (20 kDa chaperone 1)	1.57
emb|CDP47983.1|	Peroxiredoxin/Alkyl hydroperoxide reductase subunit C/Thioredoxin peroxidase/Alkyl hydroperoxide reductase protein C22/General stress protein 22	1.53
emb|CDP49312.1|	FeS assembly protein SufB	0.61
emb|CDP49702.1|	Stomatin/prohibitin	0.60
emb|CDP47885.1|	Putative O-sialoglycoprotein endopeptidase	0.59
emb|CDP48273.1|	Surface layer protein B (S-layer protein B)	0.41
emb|CDP48858.1|	Surface protein with SLH domain	0.15

*Proteins were determined by using a database composed of proteome of *P. freudenreichii* CIRM-BIA 129 (downloaded from NCBI.nlm.nih.gov 2018). Ratio were calculated using the XIC methods and indicate induction (ratio > 1.5) or repression (ratio > 0.66) by NaCl.*

### Modulation of S-Layer Protein B on *P. freudenreichii* CIRM-BIA 129 Cell Wall

**FIGURE 5:**
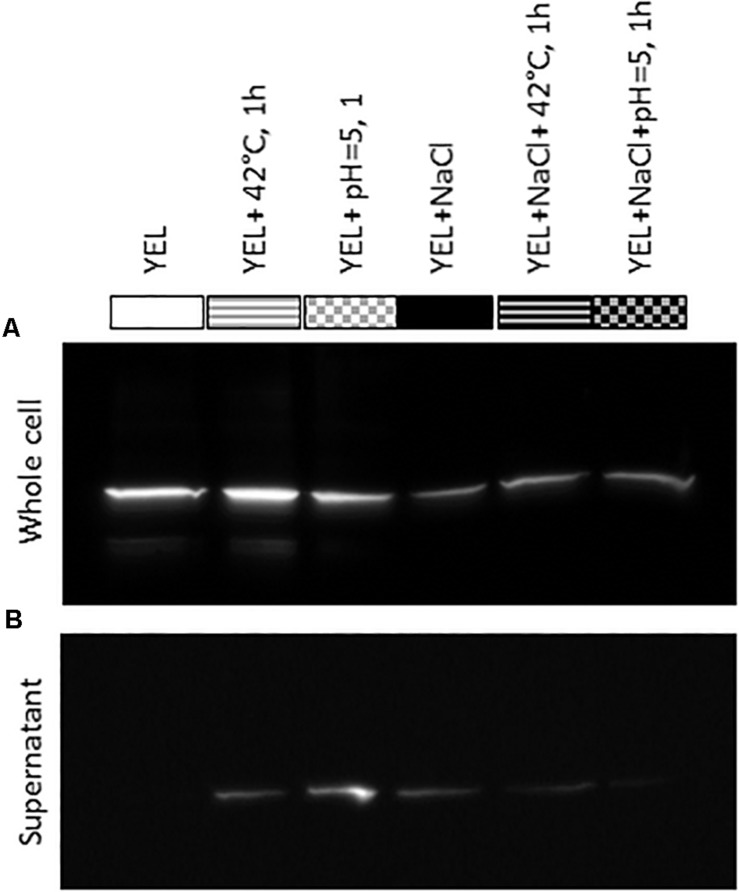
Detection of SlpB protein in *Propionibacterium freudenreichii* CIRM-BIA 129 whole-cell protein extracts **(A)** and supernatants **(B)** for cultures performed in YEL and YEL+NaCl broths, and after heat and acid adaptations. YEL: Yeast Extract Lactate. 42°C, 1 h: thermo-adaptation at 42°C during 1 h. pH = 5, 1 h: acid-adaptation at pH = 5 during 1 h.

Western blotting, using antibodies directed against *P. freudenreichii* CIRM-BIA 129 SlpB protein, allowed detection of this protein in all *P. freudenreichii* whole-cell protein extracts, whatever the growth conditions, but to different extents (Figure 5A). Indeed, the amount of detected SlpB was lower for all the cultures performed in YEL with added NaCl. Concerning the culture supernatants, no SlpB was detected in control conditions, i.e., cultures in the rich YEL medium. However, heat and acid adaptation led to the release of a part of SlpB into the growth supernatant. Less SlpB protein was detected in whole cell extracts when *P. freudenreichii* CIRM-BIA 129 was cultivated in the presence of NaCl, while traces of this protein were detected in the corresponding supernatants.

## Discussion

### Heat and Acid-Adaptations, Coupled to Osmoadaptation, Trigger Cross-Protection to Lethal Challenges

Osmoadaptation, i.e., growth in the rich YEL medium in the presence of 0.9 M NaCl, triggered enhanced viability of *P. freudenreichii* CIRM-BIA 129 during freeze drying, as reported previously for *P. freudenreichii* and for *L. bulgaricus* (Carvalho et al., 2003; Gaucher et al., 2019a). Remarkably, a mild acid adaptation, coupled to such an osmoadaptation, further enhanced *P. freudenreichii* CIRM-BIA 129 tolerance toward freeze-drying. This opens new perspectives to improve industrial production of this probiotic. However, osmoadaptation, coupled or not to heat or acid-adaptation, dramatically reduced *P. freudenreichii* CIRM-BIA 129 tolerance toward the digestive stresses mimicked by acid and bile salt challenges. By contrast, acid adaptation reportedly increases acid tolerance in another strain of *P. freudenreichii* and in *L. casei* (Jan et al., 2000; Broadbent et al., 2010). Moreover, heat adaptation increases bile salts and acid tolerance in *P. freudenreichii* (Jan et al., 2000, 2002; Leverrier et al., 2004). In the present study, heat adaptation alone increased *P. freudenreichii* CIRM-BIA 129 viability during heat challenge, in accordance with the increased thermotolerance of heat-adapted *P. freudenreichii* (Anastasiou et al., 2006), *L. paracasei* and *Lactobacillus kefiranofaciens* to heat shock (Desmond et al., 2001). To our knowledge, the present report is the first describing optimal tolerance toward freeze-drying, as a result of osmoadaptation combined with mild acid pretreatment.

### Modulation of the Membrane Fatty Acid Composition Increases the Viability During Freeze-Drying

Osmoadaptation strongly increased the saturated/unsaturated fatty acids ratio in the membrane of *P. freudenreichii* CIRM-BIA 129, as reported for *L. casei* (Machado et al., 2004). Heat adaptation reportedly decreases this ratio in the Swiss-type cheese starter *Lactobacillus helveticus* (Lanciotti et al., 2001), while not in *P. freudenreichii* CIRM-BIA 129 in the present study. Heat or acid-adaptation modulated the increase of the ratio saturated/unsaturated triggered by osmoadaptation. The ratio SFA/BFA (BFA: Branched fatty acid) was not significantly altered whatever the growth and treatment conditions. Osmoadaptation led to enhanced fatty acid chain length. Indeed, salt addition and acid adaptation, but not thermal adaptation, increased the C15-C22/C10-C14 ratio. Thus, *P. freudenreichii* CIRM-BIA 129 responded to environmental stresses by modulating its membrane fluidity via the saturated/unsaturated ratio, with a profound increase of anteiso-C15:0, the main membrane fatty acid in *P. freudenreichii* (Thierry et al., 2011) and known to play a key role in bacterial stress adaptation (Annous et al., 1997). Anteiso-C15, a fatty acid abundant in halophilic bacteria (Guan et al., 2018; Jang et al., 2018; Ding et al., 2019), is accordingly increased under hyperosmotic conditions in halotolerant Gram-positive bacteria such as *Planococcus* sp. (Miller, 1985), *Staphylococcus epidermidis* (Komaratat and Kates, 1975), and *S. aureus* (Kanemasa et al., 1972).

### Acid Adaptation, Coupled to Osmoadaptation, Increases Compatible Solutes Accumulation

*Propionibacterium freudenreichii* CIRM-BIA 129 accumulated in this study high amounts of glycine betaine during osmoadaptation, as already described (Gaucher et al., 2019a). Glycine betaine is an important compatible solute accumulated by different bacteria such as *L. plantarum* and *Lactococcus lactis* during osmoadaptation (Glaasker et al., 1996; Romeo et al., 2003). Only limited amounts of glutamate and trehalose were accumulated by *P. freudenreichii* CIRM-BIA 129 during osmoadaptation. Glutamate is a key molecule of the primary osmoregulation response (Csonka and Hanson, 1991) and is accumulated by *L. plantarum* (Glaasker et al., 1996; Kets et al., 1996). It should be noted that the rich YEL medium, composed of yeast extract, lactate and peptone, contains non-protein nitrogen (12.4 g), including glutamate and glycine betaine. The *P. freudenreichii* genome allows prediction of at least two glycine betaine import systems, i.e., OpuCA and an ABC-type choline/glycine betaine transporter (see below). Accordingly, *P. freudenreichii* was previously shown to accumulate glycine betaine in hyperosmotic conditions (Boyaval et al., 1999). Trehalose is accumulated in many different conditions such as osmotic, acid, oxidative and cold stresses (Cardoso et al., 2004, 2007; Thierry et al., 2011; Huang et al., 2016b). Trehalose can be synthetized by the OtsA-Otsb and TreS pathway previously described in *P. freudenreichii* (Cardoso et al., 2007). Accumulation of these three osmoprotectants, in response to osmotic constraint, is thus a general adaptation mechanism (Csonka and Hanson, 1991). Interestingly, acid adaptation further enhanced osmoprotectants intracellular accumulation and particularly that of glycine betaine. This enhanced accumulation is in accordance with the enhanced tolerance toward the freeze-drying challenge. To better address mechanisms responsible for such an adaptation, a whole-cell proteomic differential analysis was thus undertaken.

### NaCl Induces Major Changes in *P. freudenreichii* CIRM-BIA 129 Proteome, While Acid Adaptation Down-Regulated Part of the NaCl Stress Proteome

Osmoadaptation triggered major modifications in the whole-cell protein content of *P. freudenreichii* CIRM-BIA 129, as illustrated by the heatmap in Figure 4. The patterns corresponding to YEL and to YEL+NaCl exhibit numerous differences, while acid adaptation had little impact on the proteome. The NaCl stress proteome included many proteins involved in amino acid transport and metabolism. In particular, the ABC-type choline/glycine betaine transporter (emb|CDP49472.1|) and the ATP-binding protein OpuCA of glycine betaine/carnitine/choline ABC transporter (emb|CDP47878.1|) were in higher abundance, in accordance with the observed glycine betaine accumulation in the presence of salt (0.9 M NaCl). Different proteins such as a polar amino acid ABC transporter, an aminopeptidase and enzymes involved in amino acid biosynthesis were also enhanced and this shall lead to an enhanced intracellular pool of amino acids. Prephenate dehydrogenase (emb|CDP48583.1|) and L-asparaginase (emb|CDP49146.1|), which liberate ammonia/ammonium from amino acids, take part in intracellular pH homeostasis. L-asparaginase has already been reported as a stress protein in *Salmonella typhimurium* (Sonck et al., 2009).

About proteins involved in the transport and metabolism of carbohydrate, proteins corresponding to the utilization of alternative carbon sources are induced, including Myo-inositol catabolism IolB protein (emb|CDP49182.1|), as it happens during unfavorable conditions in *Corynebacterium glutamicum* (Chen et al., 2019). The pentose phosphate pathway, including the phosphoketolase pyrophosphate (emb|CDP48739.1|) and the ribose-5-phosphate isomerase 3 (emb|CDP49834.1|), is also enhanced. Stress-induction of this pathway, involved in intracellular homeostasis, has already been described for propionibacteria (Gaucher et al., 2019a), for bifidobacteria (Sanchez et al., 2007) and for bacilli (Goswami et al., 2018). By contrast, PTS (PhosphoTransferase System, emb|CDP49099.1|) is reduced by NaCl as in *Listeria monocytogenes* (Bae et al., 2012), and so is glycolysis (fructose-bisphosphate aldoase 1, emb|CDP48902.1|) and glycogen utilization (glycogen debranching enzyme GlgX, emb|CDP47629.1|).

Concerning lipid and transport metabolism, the induction of inositol-1-phosphate synthase (emb|CDP49767.1|) and of acyltransferase PslC (emb|CDP49388.1|) indicates enhanced biosynthesis of phospholipids, in accordance with the observed effect of salt on membrane fatty acids. These proteins are also involved in stress response in *Serratia plymuthica* and *C. glutamicum* (Nurlinawati et al., 2015; Chen et al., 2019).

Concerning cell wall, enzymes involved in peptidoglycan synthesis, such as UDP-N-acetylmuramate-L-alanine ligase (emb|CDP49363.1|), which adds short polypeptides to UDP-acetylmuramic acid, are enhanced. So is the cell division protein FtsI (emb|CDP49370.1|). By contrast, the S-layer-type proteins anchored into the cell wall via SLH domains, i.e., SlpB (emb|CDP48273.1|) was reduced in the presence of salt, in accordance with the western blot analysis (Figure 5) showing reduce SlpB. SlpB is known to be involved in immunomodulation by *P. freudenreichii*, with an anti-inflammatory effect (Deutsch et al., 2017). By contrast, *P. freudenreichii* CIRM-BIA 129 surface protein SlpA (emb|CDP49065.1|) was enhanced in the presence of NaCl, in accordance with a report showing an impact of osmotic stress on the expression of S-layer proteins in *L. acidophilus* (Palomino et al., 2016).

Adaptation to the osmotic constraint clearly enhanced the amount of general stress proteins involved in protein repair or turnover. This includes chaperones such as the three paralogs of Hsp 20 in *P. freudenreichii* CIRM-BIA 129, which protect other proteins from denaturations, as well as the two paralogs of GrpE, which prevent the aggregation of stress-denatured proteins. NaCl adaptation also enhanced proteins involved in oxidative remediation like thioredoxin (emb|CDP49795.1| and emb|CDP49048.1|), thiol peroxidase (emb|CDP49617.1|) and peroxiredoxin (emb|CDP47983.1|). In addition, the enhanced SmpB protein belongs to the superfamily of SsrA-binding proteins, which are RNA-binding proteins involved in translational surveillance (Moore and Sauer, 2007).

Acid adaptation, after osmoadaptation, did not induce important additional changes in *P. freudenreichii* CIRM-BIA 129 proteome, compared to osmoadaptation *per se*. Only eight proteins were repressed during the acid adaptation after the osmoadaptation.

## Conclusion

As a conclusion, this work highlights the positive impact of coupling osmoadaptation with a mild acid pretreatment, prior to freeze-drying. It moreover allows better understanding of adaptation mechanisms. The high accumulation of glycine betaine and the modulation of membrane fatty acid composition increased *P. freudenreichii* CIRM-BIA 129 viability during freeze-drying, opening new perspectives for efficient production of live and active probiotic propionibacteria.

## Data Availability Statement

All datasets generated for this study are included in the manuscript/Supplementary Files.

## Author Contributions

FG, KK, HR, SB, JO, SP, JJ, and VB-B performed the experiments. PM, PB, RJ, and GJ supervised the work. All the authors participated in the writing of the manuscript.

## Conflict of Interest

FG, PM, and PB was employed by company Bioprox and HR was employed by company Bba.

The remaining authors declare that the research was conducted in the absence of any commercial or financial relationships that could be construed as a potential conflict of interest.

## Supplementary Material

TABLE S1 Membrane fatty acids composition after different adaptations. The strain was previously grown in YEL and YEL+NaCl broths, and subjected to heat and acid-adaptation. Relative membrane fatty acids concentration are expressed in percentage.

TABLE S2 Proteins modulated during osmoadaptation. Proteins were determined by using a database composed of proteome of *P. freudenreichii* CIRM-BIA 129 (downloaded from NCBI.nlm.nih.gov 2018). Ratio were calculated using the XIC methods and indicate induction (ratio > 1.5) or repression (ratio > 0.66) by NaCl.
